# Hematological Profile of Hb Adana Among High School Students in Northeast Peninsular Malaysia

**DOI:** 10.7759/cureus.57353

**Published:** 2024-03-31

**Authors:** Mat Jusoh Siti Asmaa, Lee Miin Phoon, Nur Atikah Zakaria, Suryati Hussin, Rosnah Bahar, Mohd Nazri Hassan, Zefarina Zulkafli, Salfarina Iberahim, Marne Abdullah, Noor Haslina Mohd Noor, Shafini Mohamed Yusoff, Marini Ramli

**Affiliations:** 1 Department of Hematology, School of Medical Sciences, Universiti Sains Malaysia, Kota Bharu, MYS; 2 Hematology Unit, Department of Pathology, Hospital Raja Perempuan Zainab II, Kota Bharu, MYS

**Keywords:** non-deletional α-thalassaemia, hematological profile, hbe, hb constant spring, rare hemoglobin, hb adana

## Abstract

Background

Hb Adana is a non-deletional alpha (α)-thalassaemia variant resulting from mutations in α1- or α2-globin codon 59 (α^CD59^), leading to the production of unstable α-globin. Clinical manifestations can vary from silent carrier status to dependence on blood transfusions, hepatosplenomegaly, skeletal deformities, and spinal cord compression. Despite the significance of Hb Adana inheritance, studying this variant poses challenges due to the scarcity of molecular tests and the potential for routine diagnoses to be overlooked. This study aims to investigate the prevalence of Hb Adana among local high school students and assess the hematological parameters and hemoglobin analysis of Hb Adana in Malaysia.

Methodology

This retrospective study analyzed 13,721 blood samples collected from high school students participating in Malaysia's National Thalassaemia Screening Program at Hospital Raja Perempuan Zainab II (HRPZ II). Deletional α-thalassaemia was detected using multiplex gap-polymerase chain reaction (PCR), while common non-deletional α-thalassaemia was identified using multiplex amplification refractory mutation system (ARMS) PCR. Data were extracted from the HRPZ II database for analysis.

Results

Among the participants, 2327 individuals were found to have either common deletional (n=1037, 44.6%) or non-deletional (n=1290, 55.4%) α-thalassaemia. Hb Constant Spring was the most prevalent non-deletional α-thalassaemia, accounting for 53.03% of cases. Thirty-one participants (1.33%) exhibited α^CD59^α/αα, and one (0.04%) had α^CD59^α/-α^3.7^. Among the 32 subjects with Hb Adana, 87.5% were Malay, and 12.5% were Orang Asli. Additionally, seven cases of HbE/Hb Adana co-inheritance were identified. Hemoglobin levels in heterozygous Hb Adana individuals ranged from mild anemia to normal, between 95 g/L and 153 g/L. Mean corpuscular volume (MCV) and mean corpuscular hemoglobin (MCH) were approximately 73 fL and 23 pg, respectively.

Conclusion

This study delineates the distribution of α-thalassaemia mutation patterns among high school students in Kelantan, Northeast Peninsular Malaysia. Our findings indicate that Hb Adana is rare in our region and co-inheritance with an α-gene deletion results in α+-thalassaemia and with HbE, α0-thalassaemia. All heterozygous Hb Adana individuals exhibited low MCVs and MCHs.

## Introduction

Thalassaemia is a heterogeneous group of blood disorders involving hemoglobin synthesis inherited in an autosomal recessive pattern. Thalassaemia and related hemoglobin disorders have mutations in the globin gene that affect the globin chain production [1]. The classification is broadly divided into a quantitative reduction of the globin chain (resulting in thalassaemia) or a qualitative abnormal globin chain (resulting in hemoglobinopathies) produced in the marrow. Thalassaemia presents in two main forms: alpha (α)- and beta (β)-thalassaemias. Rarer variants include gamma (γ)-, delta (δ)-, epsilon (ε)-, and εγδβ-thalassaemias [2]. Regions endemic to malaria, such as tropical and subtropical areas, exhibit a high carrier rate of α-thalassaemia, estimated at approximately 20-30% [3-6]. This prevalence is particularly notable in Southeast Asian, Mediterranean, and Middle Eastern countries. Gene selection for α-thalassaemia confers protection against malaria falciparum [6,7]. However, nowadays, population migration has increased α-gene mutation frequency even in the malarial non-endemic regions. In Malaysia, the incidence of α-thalassaemia varies across studies, ranging from 4.5% to 15.8% [8-12].

α-Thalassaemia arises from either α-gene deletion or mutation, resulting in a complete absence or deficiency in α-globin chain synthesis. It can be broadly classified into deletional and non-deletional types [13,14]. While non-deletional α-thalassaemia occurs less frequently compared to deletional α-thalassaemia, the former is more commonly associated with severe clinical manifestations [15,16]. Notably, the majority of α-thalassaemia cases with severe clinical manifestations involve at least one non-deletional α-thalassaemia [17,18]. More than 70 forms of non-deletional α-thalassaemia have been identified, characterized by the insertion, deletion, or substitution of a single nucleotide in the α-gene, thereby altering α-globin chain synthesis [19]. Various processes, including mutant RNA splice site, RNA polyadenylation, poor RNA translation, generation of extended mRNA, and alterations in termination chains, contribute to the mutation's effects [1,20]. In Southeast Asia, common non-deletional types include Hb Constant Spring, Hb Adana, Hb Quong Sze, and Hb Pakse. Conversely, frequently encountered deletional α-thalassaemias encompass the 3.7 kb deletion (-α^3.7^), 4.2 kb deletion (-α^4.2^), SEA deletion (--^SEA^), and THAI deletion (--^THAI^) [15,21]. 

In Malaysia, Hb Adana ranks as the second most common non-deletional α-thalassaemia variant after Hb Constant Spring [15]. It arises from a mutation in either the HBA1 or HBA2 gene, located on the α1- or α2-globin gene at codon 59 of chromosome 16p13.3. This mutation substitutes glycine (GGC) with aspartic acid (GAC) in the HBA2-globin gene, resulting in the formation of a larger charged aspartic acid molecule, potentially compromising the stability of hemoglobin [22]. Hb Adana is characterized by low HBA2 levels, elevated Hb Bart's, increased zeta (ζ) chain, and a small amount of Hb H disease [23].

The first reported case of Hb Adana emerged in 2009, involving a 52-year-old Malay woman exhibiting clinical manifestations of thalassaemia intermedia and carrying a genotype of double heterozygous Hb Adana/-α^3.7^ [24]. Subsequent case series depicted presentations ranging from thalassaemia intermedia to hydrops fetalis, with subsequent family screenings revealing carrier states in either parent [25-27]. Heterozygous Hb Adana (single codon 59 mutation) remains asymptomatic, with the subtle production of unstable hemoglobin remaining undetected unless molecular DNA analysis is conducted [28].

The incidence of Hb Adana varies across different regions of the world. It is lower in Turkey (0.5-0.6%), China (approximately 1%), and Iran/Iraq (1-2.5%) [16,22,29-31]. Conversely, there is a higher prevalence of Hb Adana in countries such as Saudi Arabia (11.6%) and Indonesia (16%) [17,26,32]. In Malaysia, several studies have reported varying prevalence rates of Hb Adana. A study conducted by the Institute for Medical Research (IMR) and Universiti Kebangsaan Malaysia Medical Centre (UKMMC) reported prevalence rates of 2.5% and 1%, respectively [11]. Rahimah et al. found a prevalence of 0.01% Hb Adana among high school students participating in the National Thalassaemia Screening Program in Penang, Melaka, and Sabah [33].

The inheritance of non-deletional α-thalassaemia results in diverse clinical manifestations, ranging from asymptomatic silent carriers to dependency on blood transfusions, hepatosplenomegaly, skeletal anomalies, and spinal cord compression due to extramedullary hematopoiesis [28,34,35]. Homozygous Hb Adana (α^CD59^α/α^CD59^α) and compound heterozygous Hb Adana with Southeast Asian (SEA) deletion (--^SEA^/αα^CD59^) present as hydrops fetalis, which is incompatible with life [34,35]. Interactions between deletional and non-deletional α-thalassaemia mutations lead to Hb H disease, characterized by moderate to severe anemia and significant hepatosplenomegaly [18,35]. Heterozygous carriers of Hb Adana are generally asymptomatic, except during pregnancy, where they may experience severe anemia [34]. There is no specific treatment for heterologous Hb Adana because carriers may have normal hematological parameters and be asymptomatic. However, Hb Adana cases that presented with deletions, such as -α3.7 and -α4.2, manifest α-thalassaemia intermedia. Other than blood transfusion, treatment and management of Hb Adana intermedia may require iron therapy and hematopoietic stem cell (HSC) transplantation in severe cases. 

Hb Adana exhibits subtle changes in hematological profiles due to the unstable Hb variant and decreased expression of α-globin genes, which may be associated with the cellular processing of unstable mRNA. This is characterized by a reduced lifespan and red cell precipitation causing hemolysis [19]. Full blood count indices may reveal mildly hypochromic microcytic indices with normal hemoglobin levels. Hemoglobin analysis typically appears unremarkable [28]. Therefore, confirming the diagnosis of Hb Adana necessitates DNA studies with multiplex amplification refractory mutation system (ARMS) polymerase chain reaction (PCR) [35].

The inheritance of Hb Adana may negatively impact health, yet the study of the disease is limited due to potential diagnostic oversights with routine methods and sparse availability of molecular tests, rendering a correct diagnosis challenging. Exploring the possibility of utilizing hematological parameters and hemoglobin analysis studies to comprehend the disease behavior of Hb Adana is essential. Thus, identifying specific hematological features could aid in distinguishing Hb Adana carriers from other more common deletional types of α-thalassaemia.

This article has been posted on the Preprints.org server (doi: 10.20944/preprints202306.0579.v1) on June 8, 2023.

## Materials and methods

Study site

This retrospective study was conducted at the Hematology Unit, Department of Pathology of Hospital Raja Perempuan Zainab II (HRPZ II) in Kota Bharu, Kelantan, Malaysia. Ethical approval for the study was obtained from the National Medical Research Register (NMRR) Medical Research and Ethics Committee of the Ministry of Health (research code: NMRR-19-3700-52411 (IIR)) and the Human Research Ethics Committee (HREC) of Universiti Sains Malaysia (USM) (JEPeM code: USM/JEPeM/20010012).

Study population

**Figure 1:**
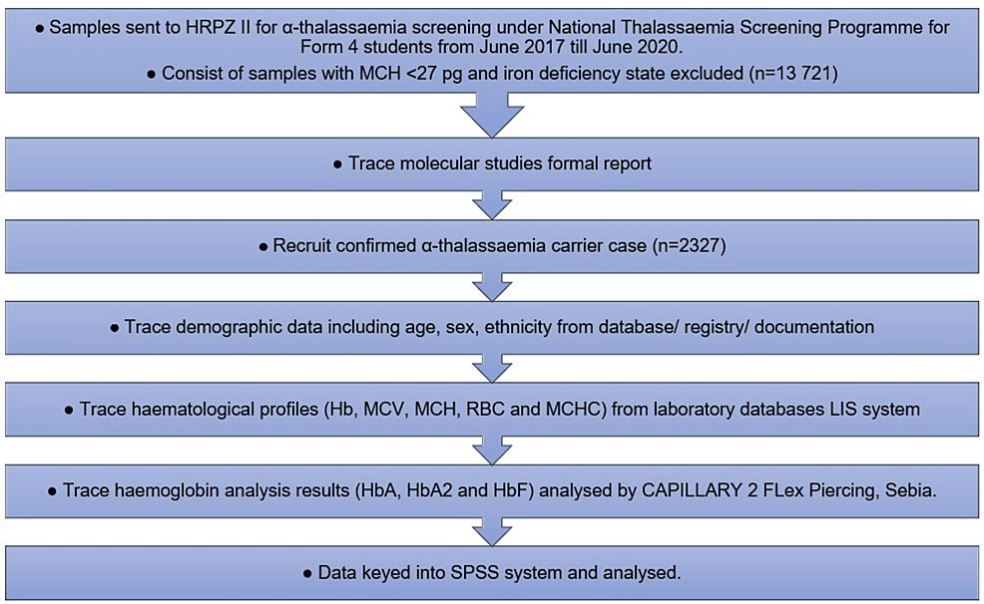
Study sampling flowchart HRPZ II: Hospital Raja Perempuan Zainab II; MCH: mean corpuscular hemoglobin; MCV: mean corpuscular volume; RBC: red blood cell; MCHC: mean corpuscular hemoglobin concentration; SPSS: Statistical Package for the Social Sciences

A total of 13,721 blood samples were screened for α-thalassaemia based on the HRPZ II registry as part of the National Thalassaemia Screening Program for high school students (16 years old), spanning three years from June 2017 to June 2020. This program was carried out at the school level by the respective district clinics in charge, where medical officers and their teams were assigned to conduct screening procedures at schools as part of health education curricula. Informed consent was obtained from the parents. The samples were sent to HRPZ II for laboratory testing. Following the National Thalassaemia Screening Program guidelines, samples with mean corpuscular hemoglobin (MCH) <27 and iron deficiency state were excluded, while the remaining samples were screened for thalassaemia carrier status through Hb analysis and molecular testing to detect common deletional and non-deletional α-thalassaemia. For molecular study, the samples were outsourced to the Molecular Laboratory of Hospital Kuala Lumpur (HKL). The study's sampling process was illustrated in a flowchart (Figure 1).

Sampling

There were 2327 subjects recruited from the convenience sampling of 13,721 subjects screened for α-thalassaemia, with a confirmed molecular diagnosis of α-thalassaemia. Based on the molecular results, samples with heterozygous Hb Adana, other non-deletional α-thalassaemia, common deletional α-thalassaemia, and compound heterozygous were included in this study. For the molecular study (outsourced), multiplex gap-PCR was utilized to detect single α-gene deletions (-α^3.7^ and -α^4.2^) and two α-gene deletions (--^SEA^, --^THAI^, --^FIL^, --^MED^, and --(α) ^20.5^) using an optimized method and primer sets published by Chong et al. [36]. The second phase single-tube multiplex ARMS PCR method was used to detect common non-deletional α-thalassaemia: initiation codon (ATG>A-G), codon 30 (ΔGAG), codon 35 (TCC>CCC), codon 59 (GGC>GAC) Hb Adana, codon 125 (CTG>CCG) or Hb Quong Sze (Hb QS, HBA2: c.377 T>C (or HBA1)), and termination codon TAA>CAA or Hb CS. The method was based on the standardized protocol published by Eng et al. [37]. Secondary data consisting of demographic data, hematological parameters, i.e., Hb, red blood cell (RBC), mean corpuscular volume (MCV), MCH, and mean corpuscular hemoglobin concentration (MCHC), and hemoglobin analysis (i.e., HbA, HbA2, and HbF) were obtained from the HRPZ II database. Subjects with incomplete documentation of hematological parameters (such as any single missed-out hematological parameter for the study, including Hb, RBC, MCV, MCH, MCHC, HbA, HbA2, or HbF) were excluded from this study. The study methods adhered to the Strengthening the Reporting of Observational Studies in Epidemiology (STROBE) initiative of reporting medical observational and epidemiological studies involving cohort, case-control, and cross-sectional designs.

## Results

Detection of common deletional and non-deletional α-thalassaemia

Our analysis detected 2327 out of 13,721 subjects screened for α-thalassaemia as confirmed α-thalassaemia carriers by molecular analysis. They consist of common deletional (n=1037, 44.6%) and non-deletional α-thalassaemia (n=1290, 55.4%). We reported 31 (1.33%) subjects with heterozygous Hb Adana and one (0.04%) compound heterozygous Hb Adana with a single α-gene (3.7kb) deletion. They were the second most common non-deletional α-thalassaemia after Hb Constant Spring (n=1234, 53%). Six subtypes under common deletional α-thalassaemia were observed, namely, heterozygous α+-thalassaemia 3.7 kb deletion (αα/-α3.7), heterozygous α+-thalassaemia 4.2 kb deletion (αα/-α^4.2^), homozygous α0-thalassaemia 3.7 kb deletion (-α^3.7^/-α^3.7^), compound heterozygous α0-thalassaemia with 3.7 kb and 4.2 kb deletion (-α^3.7^/-α^4.2^), heterozygous α0-thalassaemia SEA deletion (--^SEA^), and heterozygous α0-thalassaemia THAI deletion (--^THAI^). The distribution of α-thalassaemia among high school students in Kelantan is tabulated in Table 1.

**Table 1:** The distribution of α-thalassaemia among screened high school students in Kelantan

DNA analysis results	Frequency (n)	Percentage (%)
Non-deletional α-thalassaemia		
α^CD59^α/αα	31	1.33%
α^QZ^α/αα	24	1.03%
α^CS^α/αα	1234	53.03%
Deletional α-thalassaemia		
-α^3.7^/αα	659	28.32%
-α^4.2^/αα	43	1.85%
-α^3.7^/-α^3.7^	55	2.36%
-α^3.7^/-α^4.2^	11	0.47%
--^SEA^/αα	260	11.17%
--^THAI^/αα	9	0.39%
Compound heterozygous α^CD59^α/-α^3.7^	1	0.04%
Total	2327	100.00%

Of the 32 Hb Adana subjects, 28 (87.5%) were of Malay ethnicity, and the remaining four (12.5%) were Orang Asli (aboriginal people). The gender distribution of Hb Adana showed a slight female predominance, with 17 female and 15 male subjects. Overall, we observed a female thalassaemia carrier preponderance over males by a ratio of 1.36:1 (female-to-male ratio) in our study population. The ethnic distribution primarily consisted of Malays, with a minority of Chinese, Siamese, and Orang Asli. No subjects of Indian ethnicity were found in this study. The ethnic and gender distribution of the study population is presented in Table 2 and Table 3.

**Table 2:** Ethnic distribution of α-thalassaemia carrier among screened high school students in Kelantan

DNA analysis results	Ethnic, n(%)			
	Malay	Chinese	Orang Asli	Siamese
Non-deletional α-thalassaemia				
α^CD59^α/αα	27 (1.19)	0 (0.00)	4 (44.44)	0 (0.00)
α^QZ^α/αα	23 (1.01)	1 (3.45)	0 (0.00)	0 (0.00)
α^CS^α/αα	1223 (53.69)	6 (20.69)	5 (55.56)	0 (0.00)
Deletional α-thalassaemia				
-α^3.7^/αα	645 (28.31)	7 (24.14)	0 (0.00)	7 (63.64)
-α^4.2^/αα	43 (1.89)	0 (0.00)	0 (0.00)	0 (0.00)
-α^3.7^/-α^3.7^	53 (2.33)	1 (3.45)	0 (0.00)	1 (9.09)
-α^3.7^/-α^4.2^	10 (0.44)	1 (3.45)	0 (0.00)	0 (0.00)
--^SEA^/αα	244 (10.71)	13 (44.83)	0 (0.00)	3 (27.27)
--^THAI^/αα	9 (0.39)	0 (0.00)	0 (0.00)	0 (0.00)
Compound heterozygous				
α^CD59^α/-α^3.7^	1 (0.04)	0 (0.00)	0 (0.00)	0 (0.00)
Total	2278	29	9	11

**Table 3:** Gender distribution of α-thalassaemia carrier among screened high school students in Kelantan

DNA analysis results	Gender, n (%)	
	Male	Female
Non-deletional α-thalassaemia		
α^CD59^α/αα	15 (1.52)	16 (1.19)
α^QZ^α/αα	11 (1.11)	13 (0.97)
α^CS^α/αα	583 (59.01)	651 (48.62)
Deletional α-thalassaemia		
-α^3.7^/αα	204 (20.65)	455 (33.98)
-α^4.2^/αα	20 (2.02)	23 (1.72)
-α^3.7^/-α^3.7^	22 (2.23)	33 (2.46)
-α^3.7^/-α^4.2^	6 (0.61)	5 (0.37)
--^SEA^/αα	123 (12.45)	137 (10.23)
--^THAI^/αα	4 (0.40)	5 (0.37)
Compound heterozygous		
α^CD59^α/-α^3.7^	0 (0.00)	1 (0.07)
Total	988	1339

Seven cases of double heterozygous HbE/Hb Adana were discovered by chance while tracing DNA analysis results for subjects suspected of α-thalassaemia. The hemoglobin level for heterozygous Hb Adana varied from mild anemia to a normal level, with the lowest value at 95 g/L and the highest at 153 g/L. Their mean MCV was about 73 fL (mean 72.93±3.57 SD), and their mean MCH was less than 24 pg (mean 23.39±1.36 SD). Heterozygous Hb Adana, when co-inherited with a 3.7kb single gene deletion (α^CD59^α/-α^3.7^), exhibited a normal Hb level (124 g/L) but lower MCV (71.1 fL) and MCH (21.5 pg) compared to heterozygous Hb Adana alone. The RBC count for both Hb Adana and when co-inherited with a single α-gene was approximately 5.7x10^9^/L. Regarding hemoglobin analysis, heterozygous Hb Adana showed normal HbA2 compared with a mildly reduced HbA2 percentage in compound heterozygous Hb Adana with the 3.7kb single gene deletion (α^CD59^α/-α^3.7^) (Table 4).

**Table 4:** Hematological parameters (RBC, Hb, MCV, MCH, MCHC, HbA2, HbE, and HbF) of heterozygous Hb Adana (n=31) and compound heterozygous Hb Adana/-α3.7 (n=1) RBC: red blood cell; MCV: mean corpuscular volume; MCH: mean corpuscular hemoglobin; MCHC: mean corpuscular hemoglobin concentration

Parameters	Heterozygous Hb Adana α^CD59 ^α/αα (n=31) range	Mean (SD)	Compound heterozygous α^CD59^α/ -α^3.7^ (n=1)
Hb (g/L)	95.00-153.00	133.61 (±12.31)	124.0
RBC (×10^9^/L)	4.82-6.50	5.71 (±0.42)	5.78
MCV (fL)	58.90-79.60	72.93 (±3.57)	71.1
MCH (pg)	17.50-24.90	23.39 (±1.36)	21.5
MCHC (g/dL)	29.80- 34.20	32.06 (±0.99)	30.2
HbA (%)	96.70- 98.10	97.46 (±0.25)	97.5
HbA2 (%)	1.90-3.00	2.51 (±0.20)	2.1
HbF (%)	0.00-0.50	0.03 (±0.10)	0.4

All the cases of double heterozygous HbE/Hb Adana showed normal Hb levels with mild hypochromic microcytosis. Their MCV was consistently less than 73 fL, corresponding with the findings of heterozygous Hb Adana. The lowest MCH of HbE/Hb Adana was 21.1 pg, which was higher than the minimum value of MCH observed in heterozygous Hb Adana in this current study. The HbA2 percentage of double heterozygous HbE/Hb Adana was borderline elevated, ranging from 3.4% to 3.9%. The HbE percentage was, on average, less than 23%. There was no remarkable increase in HbF level (Table 5).

**Table 5:** Hematological parameters (RBC, Hb, MCV, MCH, and MCHC) and hemoglobin analysis (HbA, HbA2, HbE, and HbF) of double heterozygous HbE/Hb Adana (n=7) RBC: red blood cell; MCV: mean corpuscular volume; MCH: mean corpuscular hemoglobin; MCHC: mean corpuscular hemoglobin concentration

	Double heterozygous HbE/Hb Adana (n=7)						
Parameters	1	2	3	4	5	6	7
Hb (g/L)	122.0	124.0	131.0	132.0	133.0	136.0	141
RBC (×10^9^/L)	4.94	5.12	5.45	5.42	6.31	5.91	5.83
MCV (fL)	73.50	70.30	72.70	72.90	64.8	69.5	71.0
MCH (pg)	24.70	24.20	24.0	24.40	21.1	23.0	24.2
MCHC (g/dL)	33.60	34.50	33.10	33.40	32.5	33.10	34.1
HbA (%)	74.4	72.60	75.6	75.6	73.50	74.9	74.4
HbA2 (%)	3.5	3.6	3.4	3.6	4.1	3.7	3.9
HbE (%)	21.9	22.4	21.0	20.8	22.4	21.4	21.7
HbF (%)	0.2	1.4	0.0	0.0	0.0	0.0	0.0

## Discussion

This study was conducted in Kelantan, the sixth largest state in Malaysia, among the 13 states (Negeri) and three federal territories (Wilayah Persekutuan). Kelantan is located on the northeast coast of Peninsular Malaysia, bounded by Thailand to the north, Pahang to the south, Terengganu to the east, and Perak to the west, with an approximate area of 15,040 km^2^. There are 10 districts in Kelantan, namely, Kota Bharu, Bachok, Tumpat, Pasir Mas, Tanah Merah, Kuala Krai, Machang, Pasir Putih, Jeli, and Gua Musang [38]. Kota Bharu serves as the capital city of Kelantan, housing HRPZ II, the only Ministry of Health tertiary hospital in the state. All samples from the districts, including those from the National Thalassaemia Screening Program for high school students, were processed by this hospital.

Our study revealed that Hb Adana is a common non-deletional form of α-thalassaemia discovered in Kelantan after Hb Constant Spring, which is consistent with the findings of the comprehensive review on the prevalence of α-thalassaemia in Southeast Asia by Hockham et al. [15]. Interestingly, we found that the distribution of Hb Adana in our study surpassed that of a similar study conducted across three states (Penang, Melaka, and Sabah) of Malaysia by Rahimah et al. [33]. In their study, non-deletional α-thalassaemia consisted of only one Hb Adana case, 20 Hb Constant Spring cases, and two Hb Quong Sze cases [33]. Jameela et al. described a single case of Hb Quong Sze with no reported Hb Adana or Hb Constant Spring in a secondary school in Ampang, Selangor [10].

This current study found that 87.5% of Hb Adana cases were Malay, while 12.5% were Orang Asli. This finding is consistent with a previous study conducted in our neighboring country, Singapore, which reported that 93% of Hb Adana cases (12% out of 83 cases) were of Malay ethnicity among their patient registries [18]. The remaining α-thalassaemia carriers in the study primarily belonged to the Malay population, with the majority being Malay ethnicity (97.9%), followed by Chinese (1.2%), Siamese (0.5%), and Orang Asli (0.4%). According to the Department of Statistics Malaysia Official Portal, the ethnic distribution in Kelantan consists of Malay and Bumiputera (95.7%), Chinese (3.4%), Indian (0.3%), and other minorities (0.6%) [39]. This aligns with the expected findings based on Kelantan's population distribution. However, the data on α-thalassaemia carriers among Orang Asli in this study may not be representative. Orang Asli are the aboriginal people who inhabit the heart of the deepest jungle and rarely venture out of their comfort zones except under exceptional circumstances [40]. The National Thalassaemia Screening Program was conducted at the school level by district clinics and might have omitted the Orang Asli population. Most of the Orang Asli face financial and cultural restrictions, which diminish their chances of attending school, thus resulting in missed opportunities for screening [41].

There was a slight female predominance with a ratio of 1.36 to 1 (female to male) among our study population, which may explain the higher incidence of female Hb Adana inheritance compared to male Hb Adana. The study's source population was obtained from the National Thalassaemia Screening Program among high school students, with sample collection conducted by the Ministry of Health at the school level. According to 2019 data from the United Nations Educational, Scientific, and Cultural Organization (UNESCO), completion rates of secondary-level schooling were consistently higher among girls than boys [42]. This may elucidate the higher female population observed in this study, possibly due to a higher dropout rate among boys.

A previous study by Bozdogan et al. [22] exhibited hematology parameters of heterozygous Hb Adana with a mutated codon 59 at the α1-globin gene identified in Adana Province, Turkey, which were similar to our study. The results demonstrated a normal Hb level (mean 13.4 g/dL±1.0 SD, range 12.8-14.4), a low MCV value (mean 75.5 fL±2.8 SD, range 72.6-78.2), and a low MCH value (mean 25.5 pg±1.0 SD, range 24.4-26.3) [22]. However, the mean RBC count (mean 5.2x10^9^/L±0.2 SD, range 5.0-5.5) was low. Additionally, a single case of compound heterozygous Hb Adana with a 3.7kb single gene deletion (α^CD59^α/-α^3.7^) was detected, presenting with mild anemia and more pronounced hypochromic microcytosis. The study demonstrates that heterozygous Hb Adana in the Turkish population exhibited hematological parameters resembling α+-thalassaemia phenotypes and α0-thalassaemia when compounded with a single α-gene deletion. However, in the Malaysian population, heterozygous Hb Adana exhibited hematological parameters resembling only α+-thalassaemia phenotypes. Despite Hb Adana being compounded with a single α-gene deletion, it manifested α+-thalassaemia phenotypes based on hematological parameters. Another study by Singh et al. [17] in the US population reported that compound heterozygous Hb Adana with a 3.7kb single gene deletion (α^CD59^α/-α^3.7^) exhibited dissimilarities, with low Hb but equally moderately low MCV and MCH (Hb mean value (91 g/L±14 SD), MCV (mean 72.5 fL±5.8 SD, range 59.5-83.8), and MCH (mean 23.3 pg±1.7 SD, range 19.0-25.9)). Separately, a novel compound heterozygous Hb Adana that consists of two-point mutations (HBA1: c.179G>A) and codon 127 (A>T) (HBA2: c.382A>T) was reported in a Kurdish family in Iran, leading to a severe form of α+-thalassaemia. Results of hematological parameters showed abnormal indices, with the blood smear revealing hypochromia, anisocytosis, poikilocytosis, teardrops, and fragmented cells [43].

HbE is a common structural hemoglobinopathy in Asia involving a mutation of the β-globin gene that substitutes glutamic acid for lysine at codon 26 (GAG→AAG). The incidence of double heterozygous HbE/Hb Adana was unusually high based on the figures obtained, in contrast to the rarity mentioned by Achour et al., who reported the first case combination of HbE with Hb Adana in 2018 [44]. The prevalence of HbE varies between studies, ranging from 11.25% to 19.3% in Malaysia and 12.9% in southern Thailand [9,45,46]. Thus, we postulate that there is a higher chance of different inheritance combinations of thalassaemia in the Malaysian region involving α-thalassaemia with HbE. The nature of the disease for HbE shows similarities with β-thalassaemia due to a mutation that activates the cryptic splice site. It is now well known that compound heterozygosity of β-thalassaemia with α-thalassaemia reduces the imbalance in the globin chain and ameliorates the clinical phenotypes. Thus, inheritance of α-thalassaemia with HbE can decrease the globin chain imbalance as well [47,2]. The hematological parameters have been reported as normal Hb levels with lower MCV, MCH, and HbE percentages compared to the inheritance of the HbE trait [1,48].

Based on the seven cases of double heterozygous HbE/Hb Adana detected in our study, we observed relatively uniform result parameters similar to the interaction of HbE with deletional-type α0-thalassaemia (two α-gene deletions) as discussed by Fucharoen et al. in their previous study [47]. The study exhibited slightly lower mean Hb (mean 12.5 g/dL±1.4 SD), MCV levels (mean 77±5 SD), and HbE percentage (20.7±1.2 SD) for double heterozygous HbE with α0-thalassaemia [49]. It was reported that double heterozygous HbE with α+-thalassaemia showed near-normal FBC indices and a higher HbE percentage, with mean Hb (13.1 g/dL±1.4 SD), MCV (mean 88±4 SD), and HbE percentage (mean 28.5±1.5 SD) [47,50]. Our study reported normal Hb for double heterozygous HbE/Hb Adana, which aligns with the findings of the Achour et al. study, but our results showed more marked microcytosis than their study. The Achour et al. study showed normal Hb of 14.1 g/dL, MCV of 83 fL, and mild hypochromia of MCH 25.1 pg. Their percentage of HbE was 20.2%, which was also very similar to our findings [44]. Thus, heterozygous Hb Adana in our population exhibited hematological parameters that resembled α0-thalassaemia phenotypes when co-inherited with the HbE trait.

As for other types of non-deletional α-thalassaemia, double heterozygous HbE/Hb Constant Spring exhibited lower Hb levels, normal MCV, and mildly reduced MCH compared to HbE/Hb Adana in our study. Their result parameters were as follows: Hb (mean 114 g/L±14 SD), MCV (mean 80.2 fL±4.2 SD), and MCH (mean 24.6 pg±1.8 SD) [51]. We observed that the behavior of Hb Adana was quite similar to Hb Constant Spring when inherited with the HbE trait, as supported by previous studies [51].

Several limitations were noted in this study. The number of tested samples was slightly lower than expected from March 2020 until June 2020 due to the coronavirus disease 2019 (COVID-19) pandemic resulting in school closures, which halted screening work. We anticipated higher student enrollment during this period, which might have improved data analysis. Some of the DNA analyses (n=101) sent during the study period had pending results, possibly due to delayed testing caused by the COVID-19 pandemic's impact on laboratory services. The review of secondary data made it impossible to further molecularly test Hb Adana cases to identify the affected α1- or α2-globin genes. The postulation of α+-thalassaemia or α0-thalassaemia phenotypes was based on the hematological features from laboratory testing. We could not obtain documentation on the clinical phenotypes of the subjects enrolled in this study to better characterize the disease, as this study was based on convenience sampling of secondary data. The familial pattern of Hb Adana-positive cases was not evaluated due to ethical considerations, time constraints, and movement restrictions imposed by the COVID-19 pandemic. 

## Conclusions

We noted that heterozygous Hb Adana is rarely encountered in our region, primarily affecting Malay individuals. Additionally, the study identified cases of HbE/Hb Adana. It is concluded that heterozygous Hb Adana, combined with a single α-gene deletion, results in α+-thalassaemia. Co-inheritance of HbE changes the disease nature to α0-thalassaemia. Heterozygous Hb Adana subjects exhibited a range of hemoglobin levels, from mild anemia to normal, and low mean MCV and MCH. The study provides valuable insights into this rare thalassaemia variant, aiding in the better identification and understanding of this condition. Emphasizing the importance of adherence to the screening program is crucial to ensuring early detection of carrier status, which will be beneficial for genetic counseling and future spouse selection. A new generation free from the deleterious outcomes of thalassaemia complications is crucial to reducing the country's health burden.
